# Utidelone combined with radiotherapy followed by denosumab maintenance in a breast cancer patient with clivus metastasis: a long-term remission case report and literature review

**DOI:** 10.3389/fonc.2026.1790539

**Published:** 2026-04-22

**Authors:** Mengmeng Zhao, Xiaoyu Wan, Yan Ma, Ying Ge

**Affiliations:** 1 Department of Radiation Oncology, The Second Hospital of Jilin University, Changchun, China; 2 Department of Breast Surgery, The Second Hospital of Jilin University, Changchun, China

**Keywords:** breast cancer, case report, clivus metastasis, denosumab, radiotherapy, utidelone

## Abstract

Breast cancer stands as the most prevalent malignant tumor among women globally and is a significant contributor to cancer-related mortality. The distant metastasis sites of breast cancer patients were mainly the lung, liver and bone. Clivus metastasis of breast cancer is extremely rare and has been reported only in case reports. The early symptoms associated with this type of metastasis are insidious, making detection during routine examination challenging for patients with breast cancer. Previous reports indicate that the median survival for patients with clivus metastasis was only 9.4 months, and there is a lack of unified treatment standards. Some patients develop drug resistance after multiline treatment, presenting significant therapeutic challenges. In this case report, for the rare refractory disease of breast cancer with clivus metastasis, the whole course treatment mode of volumetric modulated arc therapy (VMAT) combined with concurrent chemotherapy with utidelone, followed by long-term maintenance of denosumab, was adopted. Over a follow-up period of 30 months, no recurrence was observed. This regimen combines local lesion control with high-dose radiotherapy and systemic chemotherapy with a novel epirubicin microtubule stabilizer, utidelone. Denosumab, which is used for maintenance therapy after concurrent chemoradiotherapy, is valuable for both bone protection and tumor microenvironment regulation and ultimately achieves rapid and durable clinical remission. This report provides a reference for the treatment of similar rare metastatic cases and provides important preliminary evidence for follow-up clinical research.

## Background

Breast cancer remains the most prevalent malignant tumor among women globally and is a leading cause of cancer-related deaths, posing a serious threat to women’s health. According to global statistics, breast cancer accounts for 23.8% of new female cancer cases in 2022, and 67,000 people die from this disease (1). The common metastatic sites of breast cancer are the lung, liver and bone (2). However, metastasis to the clivus is exceedingly rare and typically documented only in case reports (3–7). Early detection of clivus metastases is often challenging, as routine imaging of the skull base is not standard for breast cancer patients. Even when patients with breast cancer are regularly followed up by head MRI after treatment, early signs of clivus metastasis can be easily overlooked by radiologists and oncologists. When headache or neurological symptoms occur, clivus metastasis becomes severe. Patients with breast cancer metastatic to the clivus typically have a short survival duration. Nadeem-Tariq et al. reported that a breast cancer patient with clivus metastasis and sphenoid sinus invasion survived for only 1 month (6). In terms of treatment, evidence-based medical guidelines for clivus metastases from breast cancer are lacking, and no standardized therapeutic approach has been established. In addition, before the occurrence of clivus metastasis, breast cancer patients often have metastasis from other sites. Some patients have been treated with multiple lines of treatment and have the problem of drug resistance, which makes subsequent treatment difficult. Therefore, there is an urgent need to explore systemic treatment strategies with new therapeutic drugs, such as chemotherapy, and synergetic effects with local treatments, such as radiotherapy, to improve the prognosis of these patients.

Herein, a case of a patient who developed clivus metastasis 11 years after breast cancer surgery has been reported. The patient received concurrent chemoradiotherapy with utidelone and volumetric modified arc therapy (VMAT), followed by long-term maintenance therapy with denosumab, which resulted in a rapid, dramatic, and durable clinical and radiographic response and progression-free survival (PFS) of 30 months. This case is intended to provide an innovative and actionable whole-course management strategy for this clinical dilemma and to inform further clinical research.

## Case presentation

### Initial diagnosis and treatment

In August 2012, a 44-year-old female patient was diagnosed with breast cancer (Luminal B type; T2NxM0). She underwent modified radical mastectomy in our hospital, and postoperative pathology showed invasive carcinoma of the left breast. Immunohistochemical staining results: ER (+ ++), PR (+ +), HER2 (+), E-Cadherin(+), Bcl-2 (+), p53 (positive rate 20%), Ki67 (positive rate 30%), EGFR (-), CK5/6 (-), CK14 (-). After surgery, the patient received 6 cycles of adjuvant chemotherapy with paclitaxel and carboplatin (the specific dose was unknown). After chemotherapy, tamoxifen (10mg, orally, twice daily) was given for endocrine maintenance therapy.

### First disease progression (bone metastasis)

In October 2014, the patient presented with anterior chest pain for one month. Bone scan showed abnormal radioactive concentration in the sternum, and bone metastasis of breast cancer was considered. In order to establish the diagnosis, a sternal needle biopsy was performed, and the pathological specimen showed that the morphological features and immunohistochemical staining results supported breast cancer metastasis. Immunohistochemical staining results: ER (+, positive rate 90%), HER-2(1+), PR(+, positive rate 60%), CK7 (+), Ki67(positive rate 60%). After the diagnosis of bone metastasis from breast cancer, the patient underwent ovarian surgery for castration. Endocrine therapy was changed to anastrozole (1 mg orally, once daily) and chest pain was relieved after treatment.

### Second disease progression (bone metastasis)

In September 2015, the patient was admitted to our hospital for routine examination. Bone scan results suggested iliac bone destruction, which was considered to be caused by breast cancer metastasis. Endocrine therapy was changed to exemestane (25 mg,orally, once daily). Concurrently, she received zoledronic acid (4 mg, intravenous infusion, monthly) as anti-bone metastasis therapy, which was continued for one year. At the same time, local radiotherapy to the sternum and ilium was performed in our radiotherapy department (45Gy in 15 fractions).

### Third disease progression (multiple bone metastases)

In July 2018, the patient suffered from pain all over the body for half a month. Re-examination of bone scan showed new radioactive concentration lesions in the left 2nd and 3rd anterior ribs, the T7 and T8 vertebral bodies and the right humeral head, indicating progressive bone metastases. Given the concern of endocrine resistance, endocrine therapy was discontinued, and the mTOR inhibitor everolimus (5 mg, orally, once daily) was administered. At the same time, the patient received chemotherapy with a regimen combining paclitaxel and capecitabine (Paclitaxel 240 mg, intravenous infusion, day 1; Capecitabine 1500 mg, twice daily, orally, days 1-14) for 6 cycles. Bone pain was relieved after chemotherapy. To prevent recurrence, single-agent maintenance therapy with oral capecitabine was continued at the original dose. After 2 weeks of taking capecitabine alone, the patient developed pain and suppuration at the tips of her fingers, and capecitabine-induced hand-foot syndrome was considered. The treatment was changed to 3 courses of oral S-1 (tegafur, gimeracil, and oteracil potassium) at a dosage of 60 mg twice daily (two weeks on, one week off).

### Development of clivus metastasis and subsequent treatment

**Figure 1:**
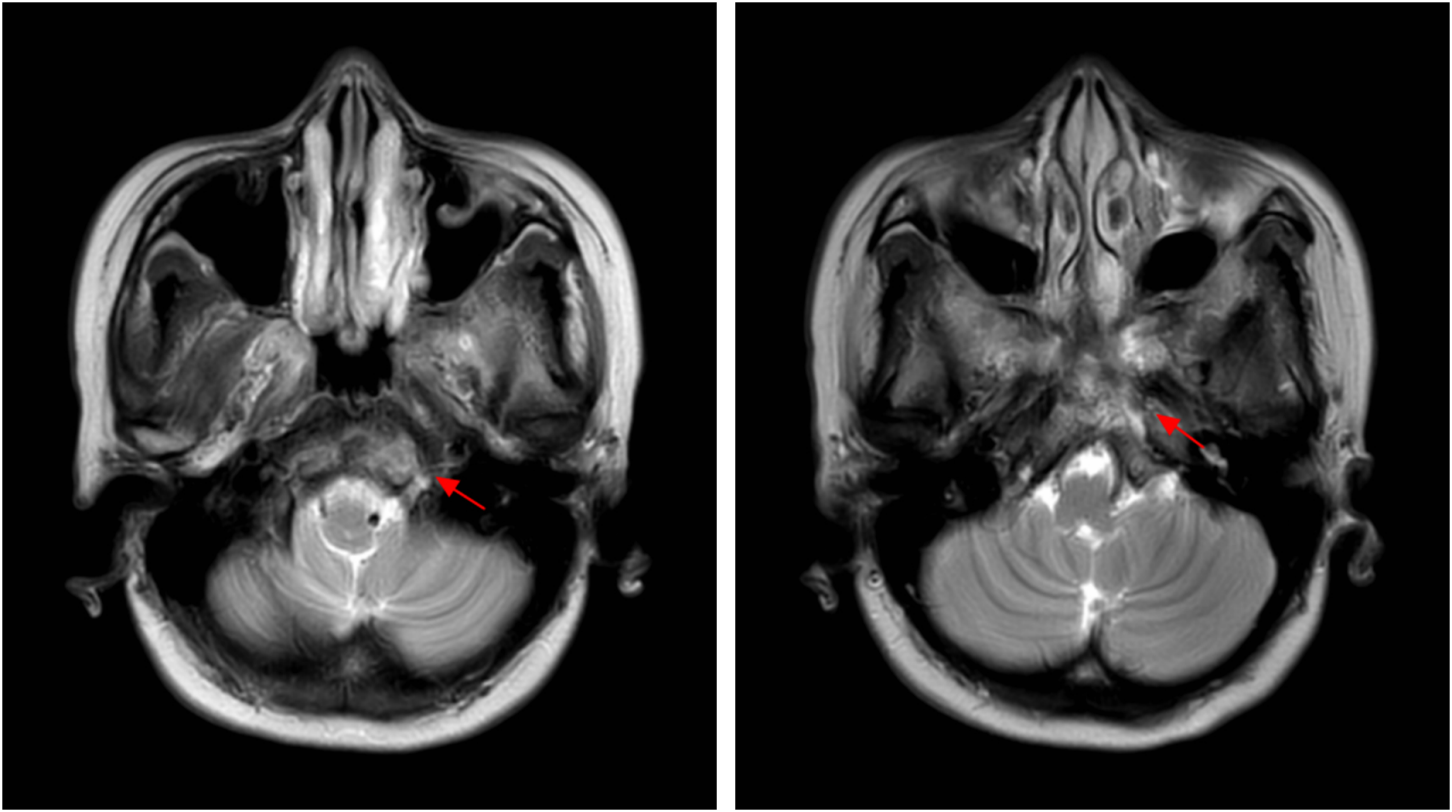
Pre-radiotherapy head MRI (T2W-MV sequence): Axial images demonstrate a well-defined hyperintense lesion involving the clivus (red arrows). The lesion measures approximately 3.3 cm in greatest dimension. No intracranial extension is observed. These findings are consistent with metastatic disease.

In April 2023, the patient presented with a two-month history of headache, which she initially managed with self-administered ibuprofen, providing some relief. The headache progressively worsened and was accompanied by diplopia for three days. The head MRI examination in the local hospital showed abnormalities in the clivus, and the metastasis was considered. Subsequently, the patient was re-admitted to our hospital for a comprehensive examination, including head MRI examination (**Figure 1**) and bone scan. Bone scan suggested the presence of clivus metastases in addition to the previous bone metastases. The results of the breast tumor marker tests were as follows: CEA 1.2 ng/mL (normal range: <5.0); CA153 35.2 U/mL (normal range: <31.3); CA125 10.6 U/mL (normal range: <35.0). Abdominal CT, chest CT and other examinations showed no obvious abnormalities. The visual analogue scale (VAS) score for headache was 3 and the ECOG score was 1. After multidisciplinary consultation, VMAT radiotherapy was given to the lesion. The prescribed radiation dose to the target volume was 60 Gy in 30 fractions (6MV X-ray, 2 Gy per fraction). At the same time, 3 cycles of concurrent chemotherapy with Utidelone (30mg/m2, intravenous infusion, days 1-5, 21 days/cycle) was initiated alongside radiotherapy. During concurrent chemoradiotherapy, symptomatic treatment included mannitol for intracranial pressure management and sustained-release morphine tablets for pain control. The patient experienced one episode of grade II myelosuppression (neutropenia) induced by chemoradiotherapy, which resolved with recombinant human granulocyte colony-stimulating factor. After chemoradiotherapy, the patient was given denosumab (120mg, subcutaneous injection, every 4 weeks) for anti-bone metastasis treatment. Concurrently, she received oral calcium carbonate (1.5 g, twice daily) and vitamin D3 (800 IU, once daily) to prevent denosumab-induced hypocalcemia.

**Figure 2:**
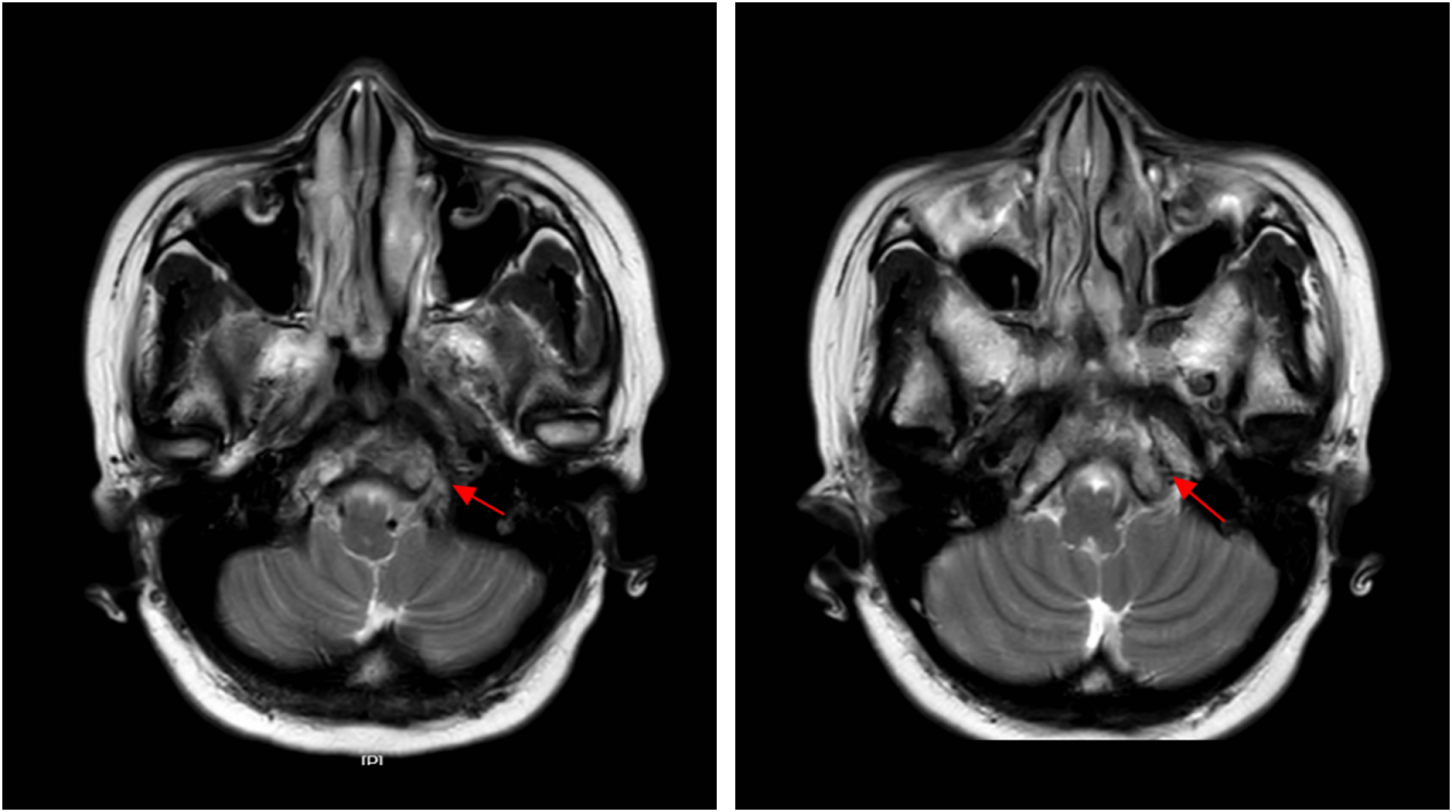
One month post-radiotherapy follow-up head MRI (T2W-mDIXON TSE sequence): Compared with pre-treatment images, the clivus lesion shows a reduction in size and a decrease in signal intensity on the T2W-mDIXON TSE fat-suppressed sequence (red arrows). No evidence of disease progression is observed.

**Figure 3:**
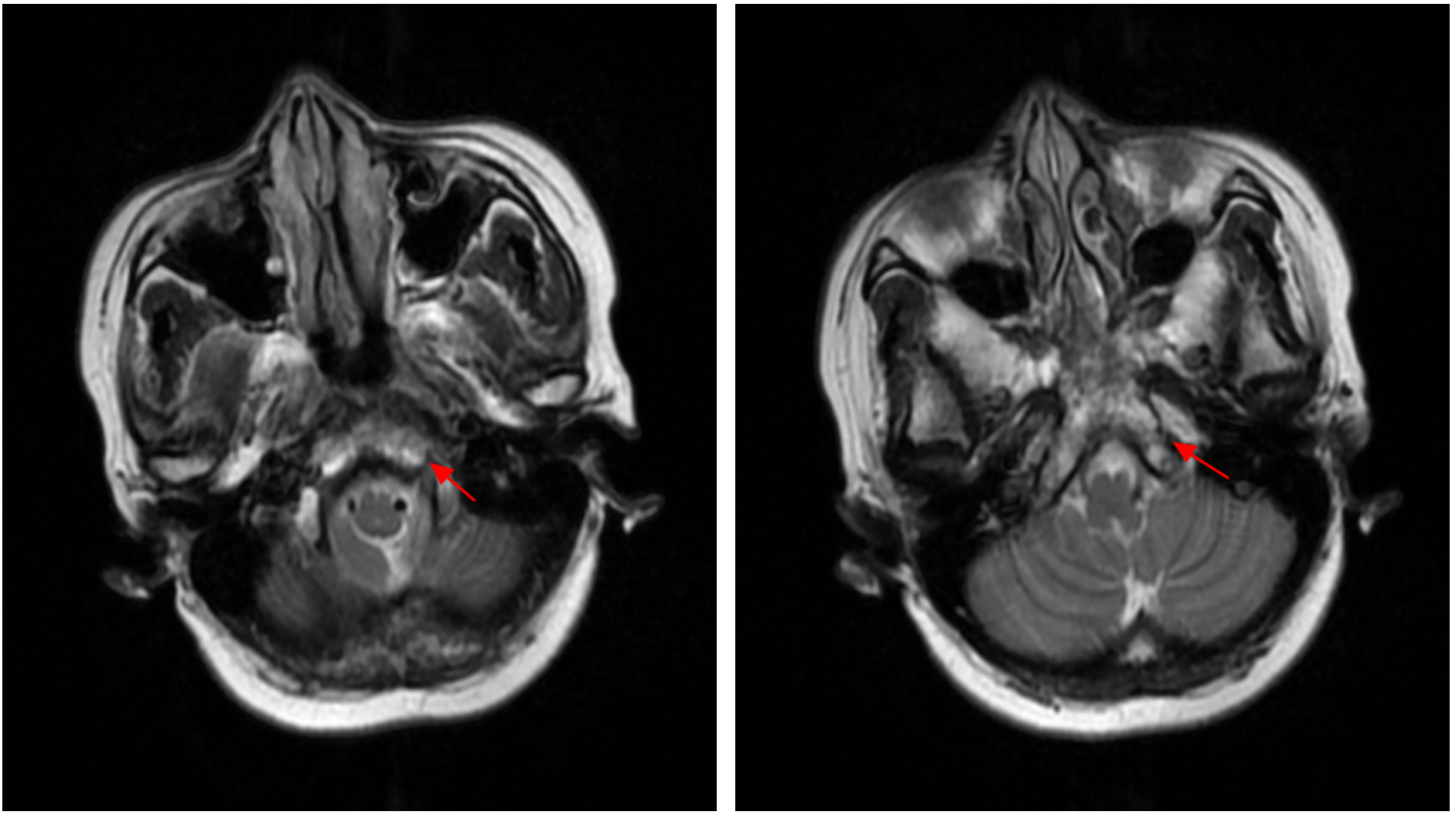
Thirty-month post-radiotherapy follow-up head MRI (T2-IDEAL sequence): Axial images demonstrate sustained regression of the clivus lesion (red arrows). The lesion size and signal intensity are reduced compared with those at one month post-radiotherapy. No evidence of local recurrence or new intracranial lesions is observed. These findings indicate durable disease control at 30 months post-treatment.

**Figure 4:**
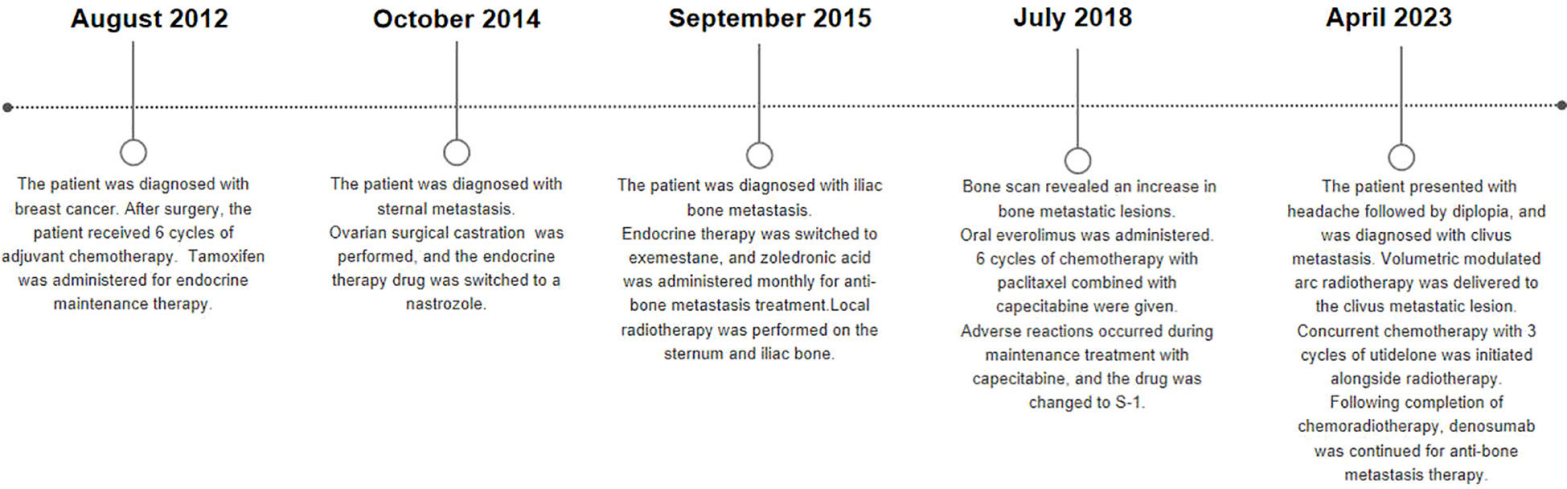
Some important time points for the treatment and follow-up of the patient after admission.

During radiotherapy, the patient’s headache was gradually relieved. After radiotherapy, the patient’s headache and diplopia disappeared, and analgesic drugs were gradually stopped. One month after completion of radiotherapy, head MRI showed that the lesions in the clivus were reduced, and there was no obvious recurrence at 30 months after radiotherapy (**Figures 2**, **3**). No long-term side effects caused by radiotherapy were observed. Breast tumor markers have been consistently within the reference range during follow-up examinations. **Figure 4** summarizes the important events of this patient through the timeline.

## Discussion

With the extended survival rates of breast cancer patients, some develop delayed distant metastases 10 years or more post-surgery, exhibiting diverse patterns of recurrence (8, 9). The occurrence of atypical metastatic sites in breast cancer patients not only presents challenges for existing examination strategies but also presents severe challenges for clinical diagnosis and treatment. Clivus metastasis is an extremely rare form of distant metastasis with a very poor prognosis. Pallini et al. reported that clivus metastasis accounted for 0.18% of all intracranial tumors treated in their hospital and 0.42% of skull base tumors (10). Jozsa F et al. systematically reviewed the literature on clivus metastasis. The inclusion criterion was articles published in English within the past 20 years as of the last search on April 12, 2021. Finally, only 58 case reports of clivus metastasis were retrieved, and metastasis at this site accounted for about 0.02% of intracranial tumors; the most common primary tumor sites were the prostate (23%), gastrointestinal tract (15%), lungs (13%), and kidneys (11%). The average survival time of these patients is only 9.4 months (11). Hematogenous metastasis, particularly via the Batson venous plexus, is considered the most likely route for clivus metastasis (12, 13). In the early stage of clivus metastasis, the symptoms of metastasis are not obvious because of small bone destruction or small masses that have not yet compressed the surrounding tissues and organs. When the tumor invades or compresses the nerve and other structures adjacent to the clivus, clinical symptoms, including progressive aggravation of headache, diplopia, and tongue deviation, occur. Diplopia is usually caused by tumor compression or invasion of the abducens nerve. Deviation of tongue extension was caused mainly by invasion of the sublingual nerve.

The clivus is a bony slope in the middle of the posterior cranial fossa in the skull base, which is formed by the fusion of the posterior part of the sphenoid body and the basal part of the occipital bone. The treatment of skull base metastasis, including that of the clivus, mainly includes surgery, radiotherapy, and chemotherapy. However, a standardized treatment protocol is still lacking. Although surgery is a more direct and effective local treatment for patients with clivus metastasis, surgery has always been recognized as one of the difficult problems in neurosurgery because of the special anatomical structure of the clivus. Moreover, surgery did not improve survival in patients with clivus metastasis. Pallini et al. collected the clinical, imaging, and follow-up data of 46 patients who underwent surgery for clivus tumors. Although these patients also underwent radiotherapy and chemotherapy, the average survival time was still 12 months (10). Chaichana et al. reported surgical results in 29 patients with skull base metastases treated at Johns Hopkins School of Medicine. Gross total resection was achieved in 34% of the cases. The median overall survival was 10 months. New postoperative complications, such as motor deficits, and new cranial neuropathy, were observed in approximately 10% of patients each (14). Radiotherapy is an effective local treatment for clivus metastases and can control tumor growth and relieve symptoms. Greenberg et al. reported 43 patients with metastatic tumors of the skull base who were treated with radiotherapy, 86% of whom experienced symptom improvement. Based on these early findings, Greenberg et al. divided patients into early treatment groups (<1 month after diagnosis) and late treatment groups (>1 month). Symptom improvement was greater in the early treatment group (92% vs 78%). In addition, the objective response rate to radiotherapy was significantly higher in the early treatment group (72% vs 28%) (15). With respect to the radiation dose of clivus metastases, previous reports have focused mainly on palliative treatment. The conventional radiation dose was concentrated between 20 Gy and 35 Gy in 5–10 fractions. The most commonly used radiation dose was 30 Gy in 10 fractions (16). For patients with controlled systemic disease and long life expectancy, Vikram et al. also suggested a radiotherapy regimen of 50 Gy in a single dose of 1.8 Gy or 2 Gy, but such a treatment dose has not been reported in the corresponding literature to date (17). Stereotactic radiosurgery is another treatment option for skull base metastases, either as a primary treatment or as a treatment for recurrence after previous radiotherapy. Minniti et al. reviewed the results of a cohort of 34 patients with skull base metastases (involving the cavernous sinus or optic nerve) treated with stereotactic radiosurgery. Patients received 25 Gy in 5 fractions. The 2-year local control rate was 72%. Clinical improvement occurred in 51% of patients after treatment (18). However, previous reports have also suggested the risk of serious complications such as cranial nerve palsy, limited mouth opening and cerebrospinal fluid leakage after radiosurgery; thus, the indications for radiosurgery should be determined, such as tumor size (19–21). Other treatment options include chemotherapy and endocrine therapy, depending on the biological characteristics of the primary disease. At present, there is no corresponding standard specific treatment plan or data. Usually, as part of systemic treatment, potentially effective therapeutic drugs are selected mainly on the basis of the biological characteristics of the primary tumor (22).

At present, there is a severe lack of experience in the treatment of clivus metastasis of breast cancer. The few literature reports focus mostly on patients who are initially treated or have received only a single local treatment. Tsunoda et al. reported a patient with clivus and sphenoid sinus metastasis 33 years after breast cancer surgery. The patient achieved remission after 44 Gy of radiotherapy combined with tamoxifen, but this patient had not previously undergone multiple lines of treatment (3). Kapoor et al. reported a patient with isolated clivus metastasis. After whole-brain radiotherapy (30 Gy), the symptoms of double vision significantly improved within 3 days, and complete remission was achieved on imaging after 3 months. However, this patient had received only adjuvant chemotherapy and endocrine therapy in the past and had not undergone multiple lines of treatment, and the follow-up period was relatively short. This case report presents a patient with luminal B breast cancer who developed clivus metastasis 11 years after multiple lines of treatment (4). Nilojan et al. reported a case of breast cancer patient with clivus metastasis at the initial diagnosis. After multidisciplinary discussion, the patient was transferred to a specialized oncology hospital for radiotherapy and chemotherapy. However, no follow-up efficacy or survival data were provided, making it impossible to assess the long-term benefits (5). For this rare metastasis, we adopted a comprehensive treatment strategy of utidelone combined with high-dose VMAT and sequential maintenance therapy with denosumab, resulting in a 30 months PFS. In terms of radiotherapy, we adopted a radical radiation dose of 60 Gy in 30 fractions. To our knowledge, this is the first time that a dose of 60 Gy has been applied to patients with clivus metastasis of breast cancer. Through VMAT technology, we achieved highly conformal wrapping of complex target areas in high-dose zones while keeping the doses received by organs at risk, such as the brainstem and optic nerve, within a safe dose range. No acute or long-term radiation injury occurred in this patient after radiotherapy. In terms of systemic treatment, this patient developed resistance to taxanes, and the options for traditional chemotherapy were limited. As a novel epirubicin microtubule inhibitor, utidelone has a different site of action than taxus drugs and still shows significant activity against advanced breast cancer after taxus resistance (23). Therefore, during radiotherapy, we adopted 3 cycles of combination chemotherapy with utidelone. We found that the patient tolerated utidelone well, with only grade 2 neutropenia occurring during chemotherapy. After concurrent chemoradiotherapy, we initiated long-term maintenance treatment with denosumab. As a receptor activator of nuclear factor-κB ligand inhibitor, denosumab is a cornerstone drug for the prevention of bone metastasis related events in solid tumors. Adding denosumab can inhibit osteoclast activity, slow down bone destruction, consolidate local treatment effect, and may delay disease progression by improving the bone microenvironment (24–26).

However, as a case report, this study has inherent limitations. Although we observed significant therapeutic responses, the lack of a control group prevented us from ruling out the influence of unmeasured confounding factors such as variations in the natural course of the disease. In addition, although the PFS has reached 30 months at present, overall survival has not yet been achieved. Further follow-up is still needed to evaluate the long-term efficacy and potential late-release toxicity.

In conclusion, this case report provides an entirely new and successful treatment modality for rare clivus metastases of breast cancer. Multidisciplinary consultation revealed that radical radiotherapy as the main treatment, induction therapy with synergistic new systemic drugs, and subsequent long-term targeted maintenance therapy can effectively result in deep and lasting remission.

## Data Availability

The original contributions presented in the study are included in the article/**Supplementary Material**. Further inquiries can be directed to the corresponding author.
